# 3D printed CNT/TPU triboelectric nanogenerator for load monitoring of total knee replacement

**DOI:** 10.1088/1361-665X/ade1ba

**Published:** 2025-06-19

**Authors:** Osama Abdalla, Mahmood Chahari, Milad Azami, Amir Ameli, Emre Salman, Milutin Stanacevic, Ryan Willing, Shahrzad Towfighian

**Affiliations:** 1 State University of New York at Binghamton, Binghamton, NY, United States of America; 2 University of Massachusetts Lowell, Lowell, MA, United States of America; 3 Stony Brook University, Stony Brook, NY, United States of America; 4 University of Western Ontario, London, Ontario, Canada

**Keywords:** 3D printing, additive manufacturing, carbon nanotubes, foam TPU printing, triboelectric nanogenerator, smart knee implant, pressure sensor

## Abstract

This study presents the development and characterization of a novel triboelectric nanogenerator (TENG) designed as a self-powered sensor for load monitoring in total knee replacement (TKR) implants. The triboelectric layers comprise a 3D-printed thermoplastic polyurethane (TPU) matrix with carbon nanotube (CNT) nanoparticles and kapton tape, sandwiched between two copper electrodes. To optimize sensor performance, the proposed CNT/TPU TENG sensor is fabricated with varying CNT concentrations and thicknesses, enabling a comprehensive analysis of how material composition and structural parameters influence energy harvesting efficiency. The 1% CNT/TPU composite demonstrates the highest power output among the tested samples. The solid CNT/TPU-based TENG generated the apparent output power of 4.1 *µ*W under a cyclic compressive load of 2100 N, measured across a 1.6 GΩ load resistance and over a nominal contact area of 15.9 cm^2^, while the foam CNT/TPU film achieved a higher apparent output power of 6.9 *µ*W measured across a 0.9 GΩ load resistance with the same nominal area. The generated power is sufficient to operate a power management and ADC circuit based on our earlier work. The sensors exhibit a stable open-circuit voltage of 320 V for the foam layer and 275 V for the solid one. Sensitivities are 80.50 mV N^−1^ ($\unicode{x2A7D} \!1600$ N) and 24.60 mV N^−1^ (> 1600 N) for foam CNT/TPU film, demonstrating the integrated sensor capability for wide-range force sensing on TKR implants. The foam CNT/TPU-based TENG maintained stable performance over 16 000 load cycles, confirming its potential for long-term use inside the TKR. Additionally, the dielectric constant of the CNT/TPU composite was found to increase with increasing CNT concentration. The proposed CNT/TPU TENG sensor offers a broad working range and robust energy-harvesting efficiency, making it appropriate for self-powered load sensing in biomedical applications.

## 1. Introduction

Total knee replacement (TKR) is one of the most commonly performed surgical procedures in the United States, with over 1 million surgeries performed annually [1, 2]. This number is expected to rise remarkably, exceeding 2.8 million procedures per year by 2040 [3, 4]. TKR effectively alleviates knee pain and enhances patient mobility. However, mechanical stresses applied on the tibial and femoral components during daily activities contribute to long-term complications, such as implant instability and loosening, which remain primarily unmonitored. While most patients experience desirable clinical outcomes post-surgery, studies show that at least 20% of patients report dissatisfaction [5–8].

Smart knee implants are crucial for monitoring a patient’s knee function, detecting potentially harmful imbalances, and initiating preventive measures to protect the patient. Therefore, by integrating force-sensing abilities, these implants can accurately measure load distribution in the knee joint, offering early intervention and eventually reducing the risk of implant failure [9]. Researchers have introduced numerous approaches to integrate these sensors into TKR implants for continuous post-surgery monitoring of daily activities and real-time force sensing. D’Lima *et al* [10] were the pioneers in developing a four-quadrant sensor system for measuring tibial forces *in vivo* after TKR. Their design integrated four separate load cells and a micro-transmitter for wireless communication, and one major limitation of this early design was its dependence on external power sources.

To improve load sensing within TKR implants, researchers investigated other transducer-based approaches. Heinlein *et al* [11] used six semiconductor strain gauges to measure loading on the tibial tray. Meanwhile, efforts were made to eliminate external power sources by developing self-powered transducers. Platt *et al* [12] investigated the feasibility of using piezoelectric ceramics (PZT) to generate electricity within knee implants. In another study, Safaei *et al* [13] and Wilson *et al* [14] integrated piezoelectric transducers within the polyethylene bearing of TKR implants to detect load imbalances and misalignments. However, a major drawback of piezoelectric materials is their lead content, a toxic substance that raises concerns regarding their long-term biocompatibility in TKR implants.

Triboelectric nanogenerators (TENGs) were introduced in 2012 and have been extensively studied as sensors and energy harvesters due to their lightweight, flexible and cost-effective [15–17]. TENGs address the growing demand for wearable electronic devices by providing a self-powered energy source, promoting the development of energy-efficient technologies, and reducing reliance on traditional batteries [18]. TENGs are capable of producing considerable electrical output even under low-frequency mechanical stimuli [19], such as human motion, making them well-suited for energy harvesting in implantable medical devices [20]. Other applications include harvesting biomechanical energy from breathing motion [21]. TENGs can also be well suited for wearable devices such as a smart belt, which harvests energy from human motion [22].

Several techniques can enhance the sensitivity and output performance of TENGs, including increasing the dielectric constant, improving mechanical durability, enlarging the effective contact area, and boosting Coulombic efficiency [23, 24]. Surface patterning is widely recognized as a practical approach to enhancing triboelectric materials’ contact area. Modifying the texture of polymer surfaces enhances charge generation and improves energy conversion efficiency [25, 26]. Common techniques to achieve this include spin coating [27], etching [28], and electrospinning [29]; however, these methods are often costly and involve complex fabrication processes.

As a promising alternative, 3D printing has emerged as a low-cost, efficient, and scalable approach for fabricating TENGs. The first 3D-printed TENG (3DP-TENG) was developed by Chen *et al* [30], who introduced an ultra-flexible device. Later, Li *et al* [31] demonstrated a flexible 3DP-TENG using viscoelastic inks, where optimized surface friction and structural design significantly enhanced energy harvesting efficiency. These advancements highlight the potential of 3DP-TENGs to improve power generation capabilities and enable broader applications in systems that rely on self-powered sensors.

Thermoplastic polyurethane (TPU) is an elastic and durable material that is among the softest 3D-printable materials that can serve as a dielectric. Moreover, the formation of microcapacitors using conductive fillers can improve the output performance of the TENG by increasing triboelectric charge storage. Conductive fillers like carbon nanotubes (CNTs) [32], MXene films [33], and graphene sheets [34] can be added to the TPU matrix in order to improve the output performance of the TENG. The CNT has high electrical conductivity and good mechanical properties [35]. Liu *et al* [36] improved the output performance of the TENG using the capacitive effect of using the CNT. They used PDMS@0.4% CNT for their simulation; the voltage reached 720 V, and the current reached 18.28 *µ*A. Nag *et al* [37] developed PDMS with multi-walled CNT as a flexible and wearable haptic TENG with a power density of 2.81 W m^−2^. Aghvami-Panah *et al* [38] investigated the electrical conductivity of TPU composites with CNT reinforcement, showing that higher CNT concentrations led to increased conductivity. In another study, Doganay *et al* [39] examined TPU composites with carbon black (CB) and silver nanowires (Ag NWs), generating 0.43 *µ*W of output power.

The biocompatibility of CNT composites remains an active area of research for implantable applications. CNT toxicity is influenced by several factors, including CNT size [40], type [41], and concentration [42]. While many studies have reported the potential toxicity of CNT-polymer composites, others have demonstrated their biocompatibility in various biomedical applications [43–45]. The long-term safety of CNT-based composites for biomedical implants still requires further investigation. To minimize potential risks, the harvester prototype must be properly sealed, and only a very low CNT concentration should be used.

In parallel with biocompatibility concerns, the functional performance of these composites under physiological conditions is also critical. While our previous studies [46–48] investigated TENGs for TKR using silicone rubber with barium titanate or PDMS as dielectric materials, those approaches required time-intensive molding and curing processes lasting 24–48 h. In contrast, the present work focuses on TPU, a widely used and processable material, and examines the effects of CNT incorporation and micro-foaming on its electromechanical performance. The use of 3D printing enables rapid, scalable fabrication using conventional desktop printers. Micro-foaming enhances triboelectric output by increasing the effective dielectric constant via microvoid formation, while CNTs contribute to performance improvement by forming microcapacitor networks, thereby increasing the overall capacitance and dielectric properties of the composite. Limited research exists on the electromechanical behavior of CNT/TPU-based TENG prototypes under pressure ranges typical of body joints—key for applications such as embedded force sensors in knee implants. This work addresses this gap by evaluating the composite for integration into TKR implants.

Our study emphasizes that a CNT/TPU-based TENG sensor was developed and embedded within a TKR package to function as a self-powered load sensor. To evaluate its performance, the proposed sensor was tested under axial compression using a sinusoidal force, simulating the cyclic peak loads experienced by knee implants during normal walking. A comparative analysis was conducted on different TPU layer thicknesses and CNT concentrations to identify the optimal load resistance in front-end electronic circuitry for maximizing power output in both solid and foam 3D-printed structures. Moreover, the experimental results demonstrated that the CNT/TPU-based TENG sensor provides stable and reliable power output.

## 2. Materials and fabrication

### 2.1. Materials

TPU (Lubrizol, Estane 2355-85ABR), characterized by a density of 1.18 g cm^−3^ and a Shore A hardness of 87, was used as the base resin. CNT/TPU masterbatch containing 15 wt% FIBRIL CNTs was provided by Hyperion Catalysis. Thermally expandable microcapsule microsphere (TEM) masterbatch (Matsumoto Yushi-Seiyaku Co., Ltd MBF-260EVA50), composed of 50 wt% TEM and 50 wt% ethylene/vinyl acetate copolymer, was used as the foaming agent and mixed with CNT/TPU at 5 wt% loading during the filament extrusion process.

### 2.2. Fabrication

**Figure 1.**
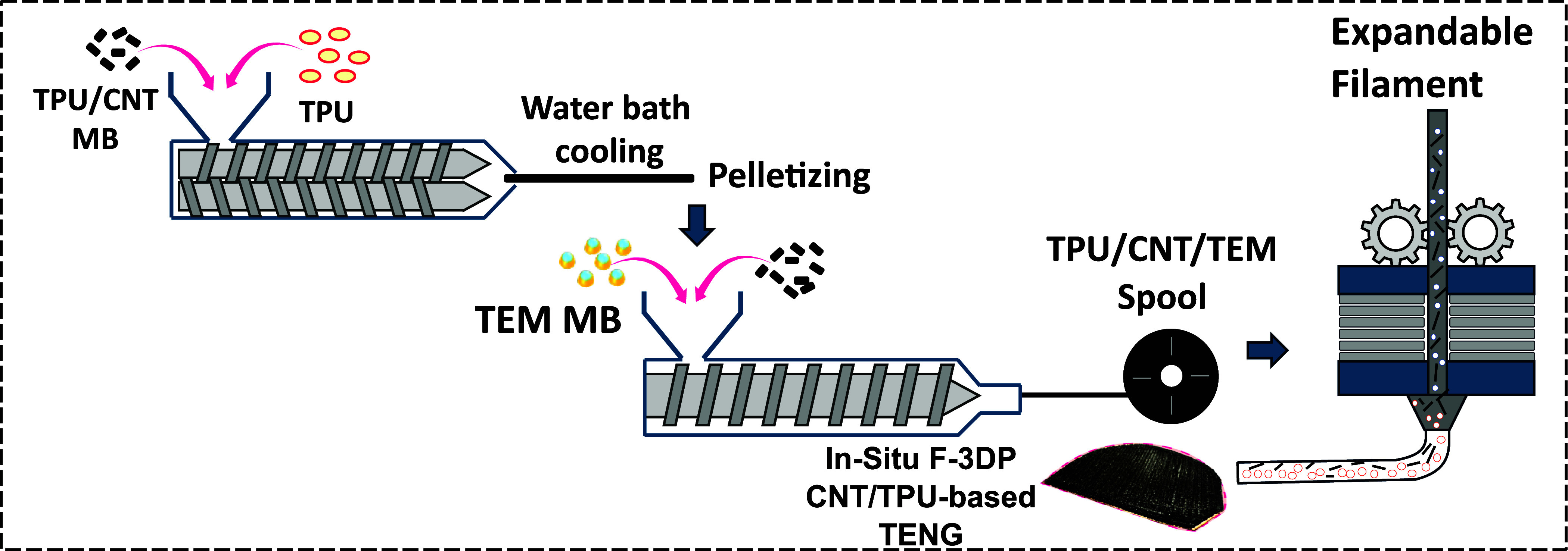
Schematic illustration of the process flow, detailing steps from compounding to filament fabrication and *in-situ* F-3DP of CNT/TPU.

Figure 1 schematically shows the process flow from compounding to filament fabrication and foam 3D printing (F-3DP) of CNT/TPU-based TENG. The solid filaments with CNT concentrations of 0, 1, 2, 3, and 5 wt%, as well as an expandable filament with 1 wt% CNT and 5 wt% TEM, were fabricated. The detailed methodology for preparing CNT/TPU nanocomposites and filaments is described in a previous study [38]. The CNT/TPU samples were also made at three different thicknesses of 0.8, 1.8, and 2.5 mm by changing the degree of foaming during printing.

Raise 3D Pro2, a commercially available 3D printer, was used to fabricate the samples based on CAD models, which were prepared and processed using IdeaMaker slicing software. The detailed relationships between the process and properties of foam samples during the 3D printing process have been discussed in previous works [38, 49, 50]. In summary, the expandable filaments undergo foaming when heated in the hot end and subjected to a reduced flow rate, which allows them to expand. Table 1 summarizes the fixed and variable printing parameters for both solid and foam samples, including the nozzle temperature. The 3D printing of CNT/TPU composites was performed using nozzle temperatures in the range 210 ^∘^C–230 ^∘^C, which falls within the recommended range for the TPU. It is worth mentioning that at these temperatures, no thermal degradation of TPU is expected, especially noting that the shear forces and residence time involved in the printing process are relatively low. The CNT dispersion was primarily established during compounding and filament extrusion processes, and no significant changes in the CNT dispersion status are expected during printing. Furthermore, while the residual heat from the freshly extruded filament does not influence CNT re-sipersion at the interlayer interface, as CNTs are embedded in viscous TPU melt, it drives the interfacial bonding between the adjacent printed layers. This thermal bonding improves the mechanical and electrical properties [38, 51].

**Table 1.** 3D printing parameters for fabrication of both solid and foam CNT/TPU.

Category	Parameter	Value
Solid printing parameters	Nozzle temperature (^∘^C)	230
	Flow rate (%)	105

Foam printing parameters	Nozzle temperature (^∘^C)	210
	Flow rate (%)	85

Fixed parameters	Nozzle diameter (mm)	0.4
	Layer height (mm)	0.2
	Print speed (mm s^−1^)	15
	Bed temperature (^∘^C)	60
	Infill density (%)	100

## 3. The working principle

**Figure 2.**
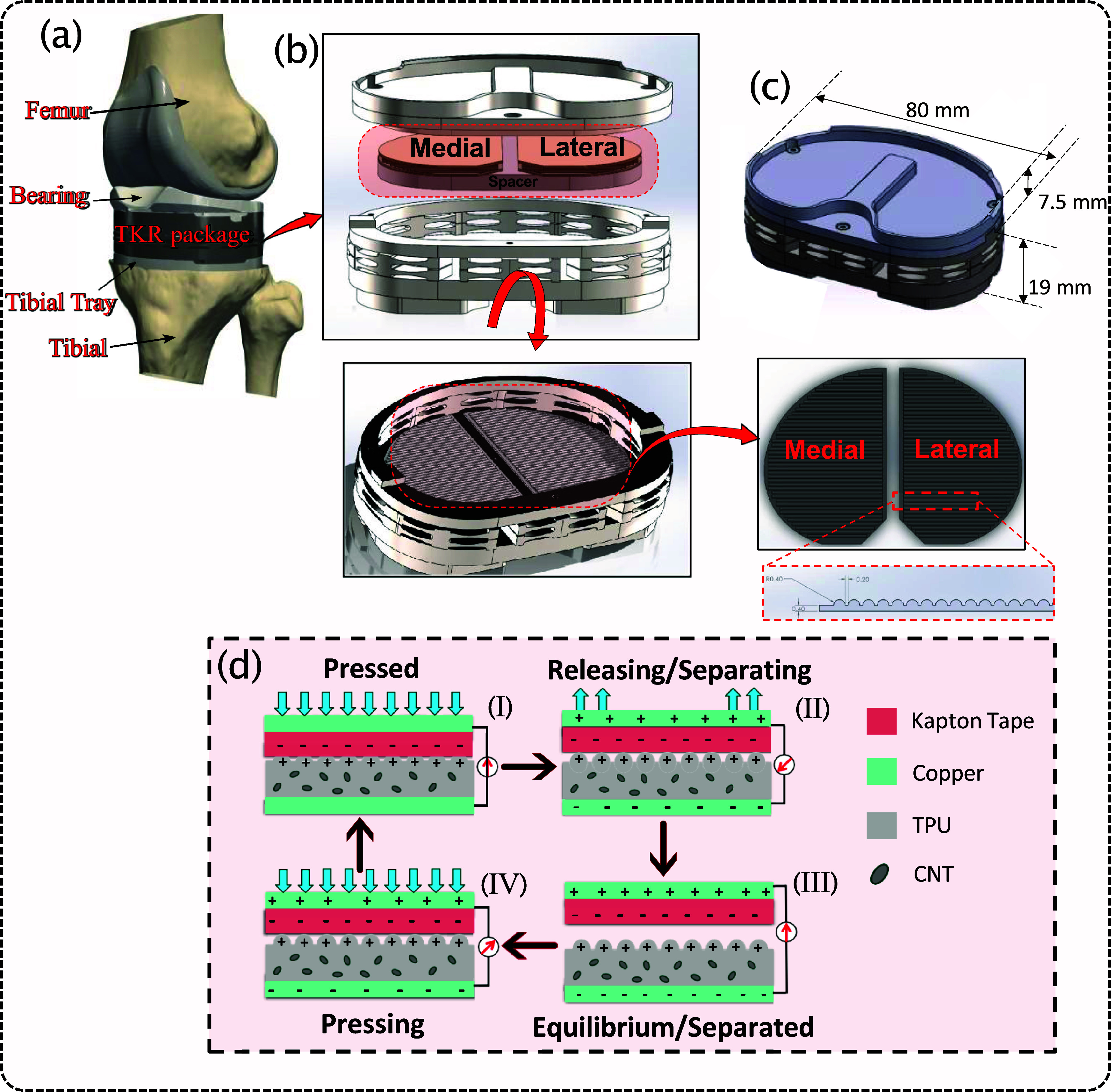
Schematic of the instrumented TKR implant with an embedded CNT/TPU-based TENG sensor for energy harvesting and force sensing as well as its working mechanism; (a) schematic of the TKR package within the knee joint. (b) Exploded view illustrating the placement of integrated sensors within the medial and lateral compartments. (c) Dimensional specifications of the TKR package. (d) Operating mechanism of the CNT/TPU-based TENG, representing charge transfer during different loading phases.

Figure 2 presents a schematic of the TKR implant integrated with a CNT/TPU-based TENG sensor, illustrating both its structure and working principle. The sensor utilizes a CNT/TPU composite and Kapton tape as dielectric materials, with copper layers serving as electrodes. To enhance performance, the CNT/TPU layer is designed with a wavelike surface pattern, which increases the effective contact area and friction when subjected to compressive forces. Additionally, incorporating CNT nanoparticles into the TPU before 3D printing improves internal friction and increases the dielectric constant, resulting in a boost of charge storing and a significant enhancement in power output [52].

When vertical pressure is applied, the wavelike CNT/TPU layer deforms, generating a restoring force that attempts to return the structure to its original shape upon load release. This deformation triggers contact electrification between the CNT/TPU and Kapton layers. The CNT/TPU surface obtains positive charges, while the Kapton tape collects negative charges, according to the triboelectric series [53], due to differences in their electron affinity. The difference in the ability to lose or attract electrons results in positive charges on the surface of CNT/TPU nanofibers and equal amounts of negative charges on the surface of Kapton tape. While the electron affinity of Kapton tape (Polyimide) is reported as 1.4–4.1 eV [54, 55], the electron affinity of TPU mixed with CNT is unknown.

Initially, when the two surfaces are in full contact, an electrostatic equilibrium is established, preventing any charge flow between the electrodes. As the surfaces separate under an external force, an electrostatic field develops between them, driving electron flow from the top electrode to the bottom electrode. This charge flow continues as separation increases, reaching a peak when the surfaces are at their maximum distance. Before full separation occurs, electrostatic induction on the electrodes maintains the charge flow. When the applied force is removed, the restoring force of the CNT/TPU layer, along with the TKR package’s spring, brings the surfaces back. This reverses the charge flow as electrons move from the bottom electrode back to the top electrode, reestablishing equilibrium. This continuous cycle of contact, separation, and charge redistribution under mechanical loading efficiently converts biomechanical energy into electrical energy.

## 4. Experimental setup

**Figure 3.**
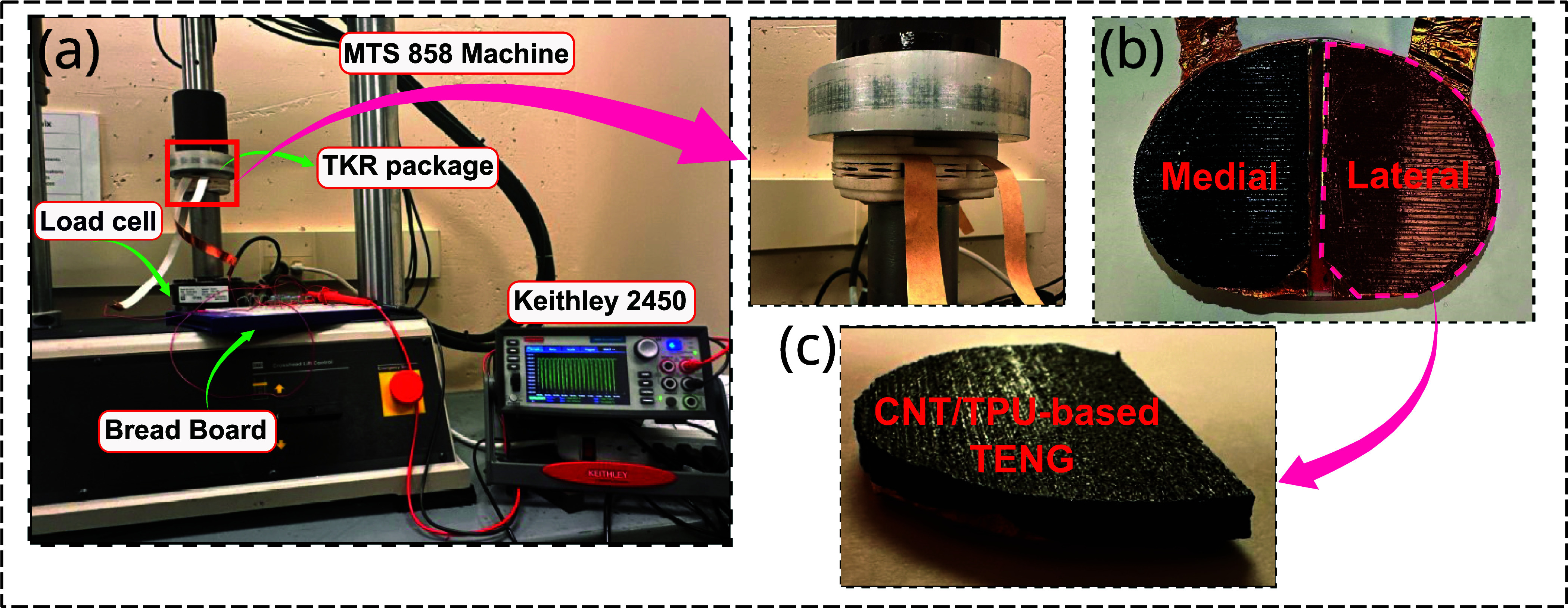
(a) Experimental setup and TKR package with integrated CNT/TPU-based TENG, (b) top view of the embedded sensors inside the package, mounted on medial and lateral compartments. (c) Side view of the CNT/TPU-based TENG sample, illustrating its wavelike surface morphology for enhanced triboelectric performance.

The fabricated CNT/TPU-based TENG sensors were integrated into the medial and lateral compartments of the TKR package, functioning as both energy harvesters and load sensors for detecting load imbalances in each compartment. The experimental setup and TKR package containing the integrated CNT/TPU-based TENG are illustrated in figure 3. A Keithley 2450 source meter was used to continuously measure the voltage and current output of the TENG during the experiment, while a 5 kN force transducer measured the applied force. Figure 3(b) shows a top view of the TENG-embedded sensors, positioned within the medial and lateral compartments inside the package. The TENG structure consists of copper tape as the conductive electrode, with the Kapton film and CNT/TPU composite film as dielectric materials. A side view of the CNT/TPU-based TENG sample is presented in figure 3(b), illustrating its wavelike (bar pattern) surface morphology. The harvester package was carefully designed to fully enclose the TENG sensors on medial and lateral compartments. As shown in figure 3(c), the prototype’s overall geometry, fabricated from biocompatible nylon using a nylon SLS 3D polymer printer (EOS), was based on the perimeter shape of a size 7 tibial tray (Triathlon, Stryker, Kalamazoo, MI). The overall height of the tibial component has been increased by 19 mm.

The MTS 858 Mini Bionix II machine was used to apply cyclic uniaxial loads on the TENG-embedded TKR package, simulating the mechanical forces exerted on knee implants during normal gait cycle. This cyclic loading induced a contact-separation mode operation in the TENG, where alternating compression and release generated triboelectric charges. Based on the experimental measurements, a potential error margin of ±50 N was considered for the force measurements recorded by the MTS machine. Human gait studies report that the knee joint experiences forces ranging from 2.5 to 3 times body weight during normal walking [56, 57]. For a 75 kg person, this corresponds to a peak force of approximately 2100 N, aligning with values reported in the literature. Most research on human motion energy harvesting has focused on normal walking, which typically occurs at a frequency of approximately 1 Hz. Accordingly, this work characterized the TENG harvester using force levels up to 2100 N and frequencies of 1 Hz.

## 5. Results and discussion

### 5.1. Solid CNT/TPU TENG

This section presents the experimental results of the TENG-based energy harvester integrated within the TKR package. The embedded solid 3D printed CNT/TPU-based TENG sensor’s energy harvesting and load sensing performance were evaluated by measuring the voltage and current outputs when a sinusoidal cyclic uniaxial force was applied, enabling the contact-separation mode operation of the TENG.

The power generation capability of the TENG depends on multiple factors, including material composition, surface contact properties, and structural dimensions. We evaluated the effect of incorporating CNT nanoparticles into solid TPU as a triboelectric material. Several CNT/TPU composite samples, each with a thickness of approximately 0.8 mm, were fabricated and attached to the medial and lateral compartments of the TKR package. The medial and lateral compartments were tested separately, with each sample printed in pairs for consistent evaluation. The samples were prepared with different CNT concentrations, specifically 0%, 1%, 2%, 3%, and 5%  by weight. To determine the optimal resistance for a power management circuit that can extract maximum power, the output voltage and current were measured across various resistive loads. These resistors, ranging from 200 MΩ to 1.6 GΩ, were connected to a breadboard circuit, and the power output was analyzed for each resistance value. Experimental results demonstrated that the highest power output was achieved at a 1% CNT concentration, whereas higher CNT concentrations led to a decrease in power generation. In contrast, a sample composed of pristine TPU (0% CNT) generated significantly lower power output, confirming the positive impact of CNT addition on triboelectric properties. The outcomes highlight that incorporating CNT into TPU remarkably improves its triboelectric properties, with 1% CNT yielding the highest power output. Beyond this concentration, excessive CNT content increases electrical conductivity, which can reduce charge retention and weaken the overall triboelectric effect.

**Figure 4.**
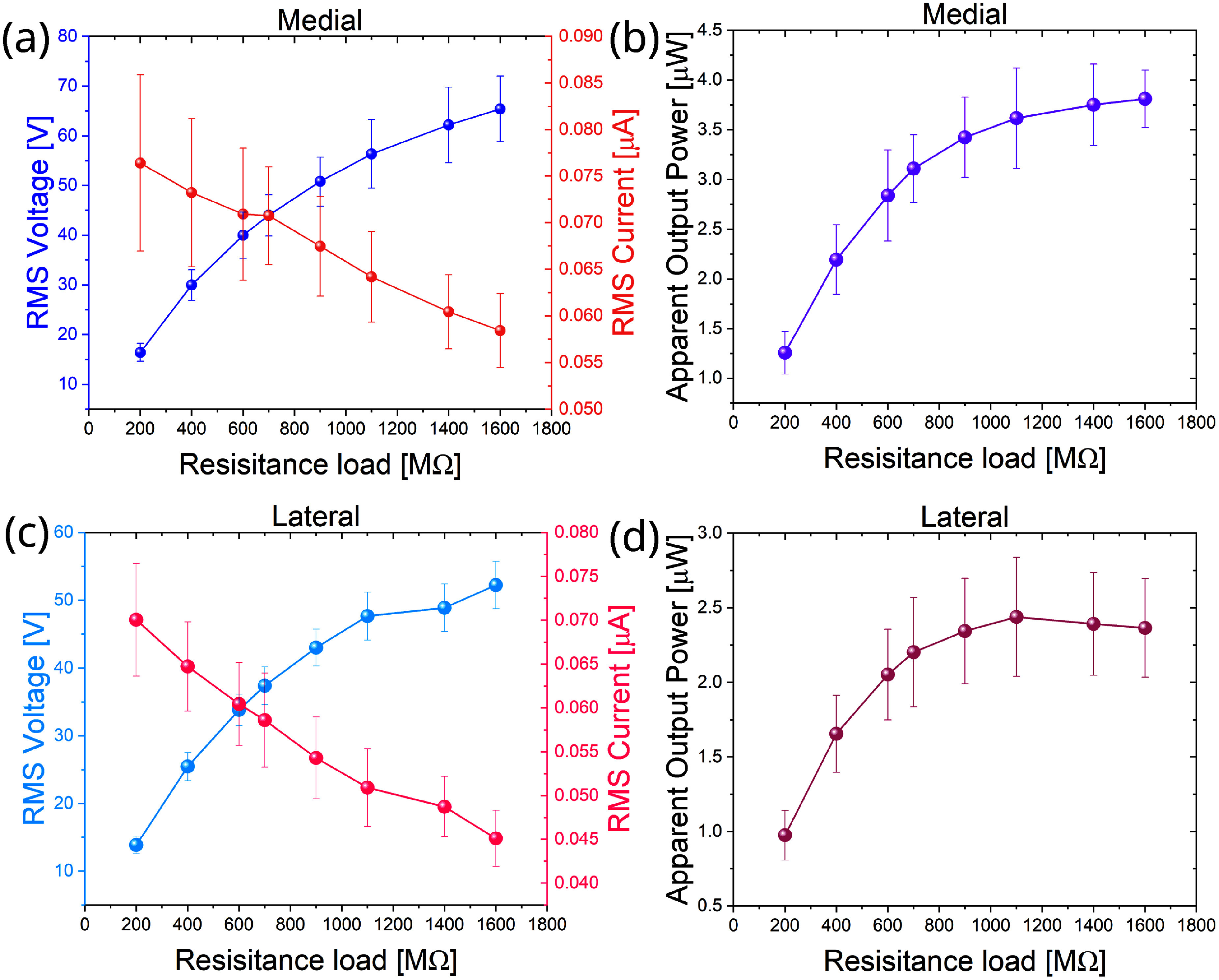
(a) and (c) error bars for RMS voltage, current, and resistors for medial-TENG and lateral-TENG at 2100 N axial compression forces, respectively. Where both are for 1% content CNT in a solid 3D printed TPU (b) and (d) error bars for the apparent power outputs for the medial and lateral sensors, respectively.

The RMS voltage and RMS current measured across varying resistance loads for the medial compartment under a 2100 N sinusoidal cyclic compression force are illustrated in figure 4(a). The experiment was repeated three times, revealing variations in the measured values. The data demonstrate a clear inverse relationship between voltage and current, which agrees with Ohm’s law: as the resistance load increases, RMS voltage increases, while RMS current decreases. A similar trend is observed in figure 4(c) for the lateral compartment, demonstrating the consistent behavior of the TENG in both compartments.

The power outputs for both load sensors on the medial and lateral compartments were evaluated by analyzing apparent output power across different resistances. Figure 4(b) displays the apparent output power of the medial compartment, showing a slow increase in power as resistance increases, with the highest power output recorded at 1.6 GΩ, reaching approximately 4.1 *µ*W. Similarly, figure 4(d) shows the apparent output power of the lateral compartment, where the maximum apparent power reached 3.1 *µ*W at 1.6 GΩ. Each data point in figures 4(a)–(d) includes error bars, representing the standard deviation of the multiple trials. The results indicate consistent power generation trends in both compartments, but with a higher apparent power output in the medial compartment (4.1 *µ*W) compared to the lateral compartment (3.1 *µ*W).

To examine the difference in the output performance between the medial and lateral compartments, the thickness of the CNT/TPU TENG layers was measured using an optical profiler (Keyence VKX 3000). The wavelike surface pattern thickness was measured at 16 different points for each sample. We found that the medial compartment had an average thickness of approximately 480.724 *µ*m, while the lateral compartment had an average thickness of around 536.188 *µ*m. The measurements were taken from the top of the pattern to the flat surface of the CNT/TPU material. This difference in thickness directly affected the voltage and power generation of the two compartments.

As the thickness decreases, the air gap increases, and the capacitance decreases. That would lead to a higher output voltage of the TENG and, consequently, higher power output as seen in figure 4(b) compared to part (d). The value of the capacitance *C* depends on the thickness of the air gap *d*, as in the equation $C = \frac{\varepsilon_0~\cdot A}{d}$ where *ε*_0_ is the permittivity of free space and *A* is the plate surface area. The decrease in capacitance C at constant charge *Q* leads to higher voltage as in $ V = \frac{Q}{C} $. The thickness variation is because of slight inconsistencies in the 3D printing process. This manufacturing defect directly influenced the charge storage capacity and voltage output, leading to the observed higher power generation in the medial compartment compared to the lateral compartment. This non-uniformity originated from the fabrication process, where the CNT/TPU composite was prepared by mixing the CNTs with the TPU matrix and then extruded into a filament for 3D printing, as mentioned in the materials and fabrication section. Due to difficulties in maintaining a uniform filament thickness during extrusion, the flow rate of the filament through the printer’s nozzle was inconsistent. This inconsistency resulted in a thickness deviation over complex geometries like wavelike patterns, while all other printing parameters are constant.

### 5.2. Foam CNT/TPU TENG

As *in situ* foam 3D printing continues to advance, the integration of nanocomposite foams with additive manufacturing provides a promising opportunity to improve the performance of printed structures by combining the advantages of foaming technology and nanocomposite materials. This approach has the potential to significantly improve the mechanical, electrical, and triboelectric properties of 3D-printed materials. However, research on the additive manufacturing of nanocomposite foams remains limited, and there is still a lack of comprehensive studies on the interaction between nanomaterials, foaming mechanisms, and the printing process, as well as their combined influence on the final properties of 3D-printed nanocomposite foams. Despite the growing interest in this field, only a few studies have studied this subject in depth. Aghvami-Panah *et al* [38] investigated the differences between foam and solid CNT/TPU composites, concluding that foam TPU shows higher electrical conductivity at lower CNT concentrations. We fabricated foam CNT/TPU-based TENG samples with 1% CNT content and tested them under the same experimental conditions.

**Figure 5.**
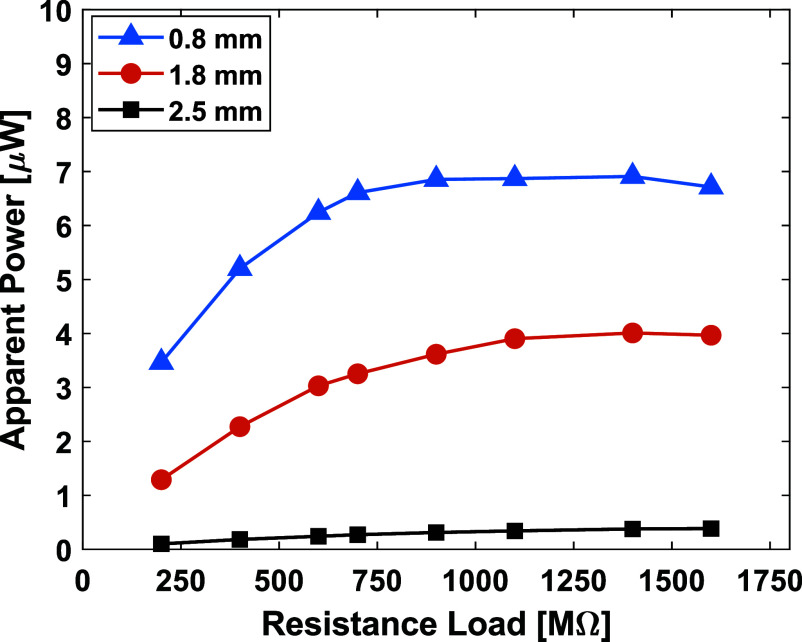
Apparent power and different resistances for medial-TENG at 2100 N axial compression forces for 1% CNT content in foam 3D-printed TPU at different thicknesses.

To evaluate the effect of dielectric thickness, foam samples with different thicknesses were 3D-printed and tested under a 2100 N sinusoidal cyclic uniaxial compression force. As shown in figure 5, the findings indicate that the apparent power output increases with dielectric thickness. The lowest power output was achieved for the 2.5 mm thick sample, which generated approximately 0.38 *µ*W at high resistance loads. Conversely, the thinnest sample (0.8 mm) exhibited the highest power output, which generated around 6.9 *µ*W at 900 MΩ. This outcome aligns with previous studies, where decreasing dielectric thickness enhances charge storage capacity, leading to higher energy conversion efficiency. Notably, our group previously reported that at least 5.35 *µ*W is required to drive a power management circuit [58]. Since the maximum recorded power output in our study is 6.9 *µ*W, the foam CNT/TPU TENG can successfully power an ADC circuit.

**Figure 6.**
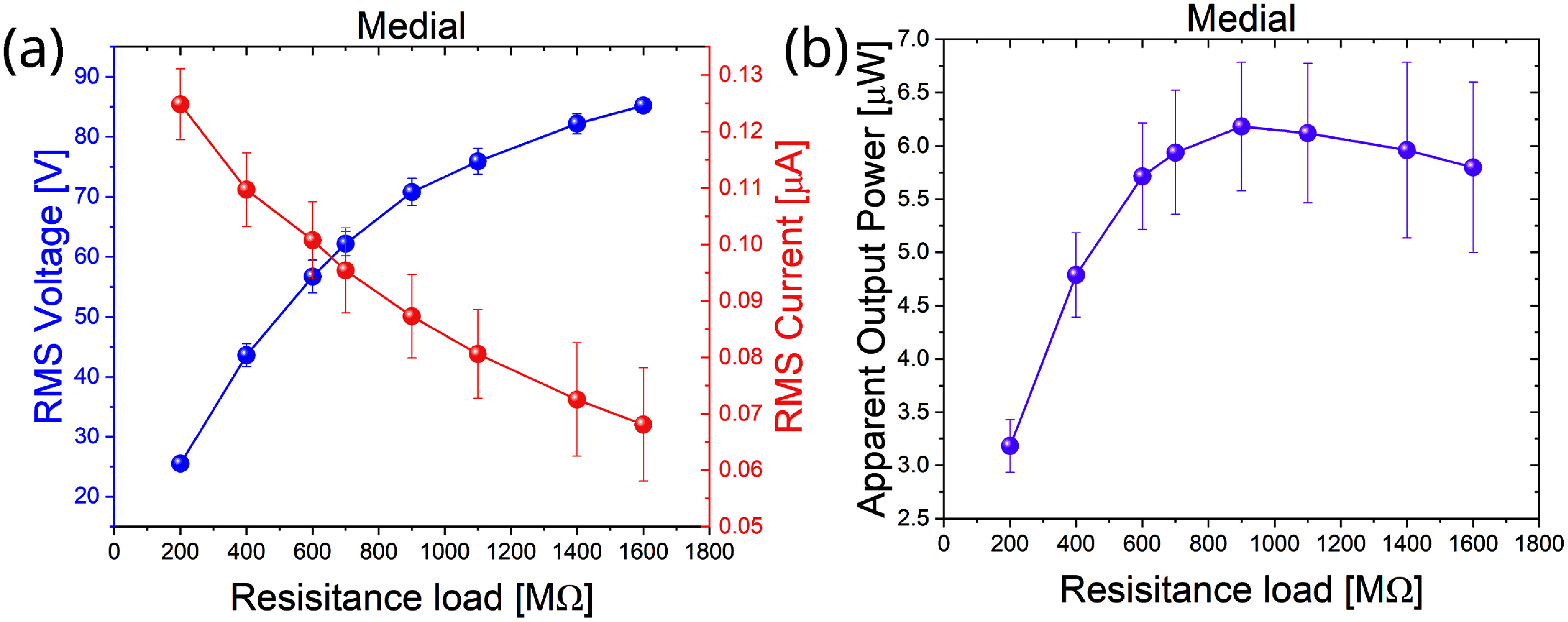
(a) and (b) RMS voltage and current and apparent power with different resistors for 3 tests were done for foam CNT/TPU at 2100 N axial compression load.

To further elaborate on the performance of foam TPU, we illustrate the RMS of voltage and current and calculate the apparent power for the medial compartment (figures 6(a) and (b)), where error bars indicate its variation. The foam TPU sample with 1% CNT was tested three times to ensure reproducibility. The maximum RMS voltage, current, and power were 85 V, 0.13 *µ*A, and 6.9 *µ*W, respectively.

**Figure 7.**
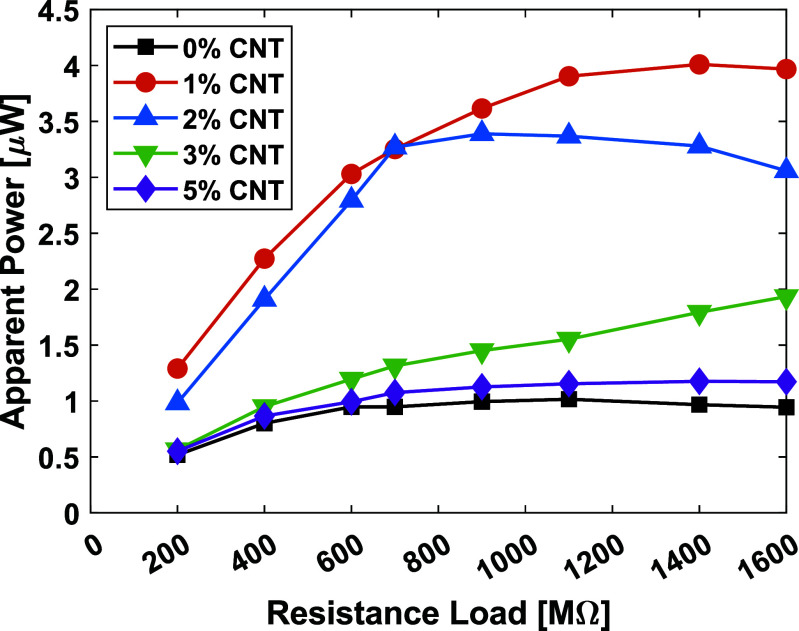
Apparent power and different resistances for medial-TENG at 2100 N axial compression forces for different CNT content in solid 3D-printed TPU.

To further analyze the effect of CNT on power generation, we measured the apparent power at different CNT concentrations in solid TPU versus resistance loads, see figure 7. The data confirms that 1% CNT concentration generates the highest power output. Increasing CNT content by more than 1%  leads to a decline in power generation. Moreover, pristine TPU shows significantly lower power output, verifying that CNT addition enhances triboelectric properties because of the charge-trapping capability. These results show the importance of optimizing CNT concentration for maximum energy harvesting efficiency.

**Figure 8.**
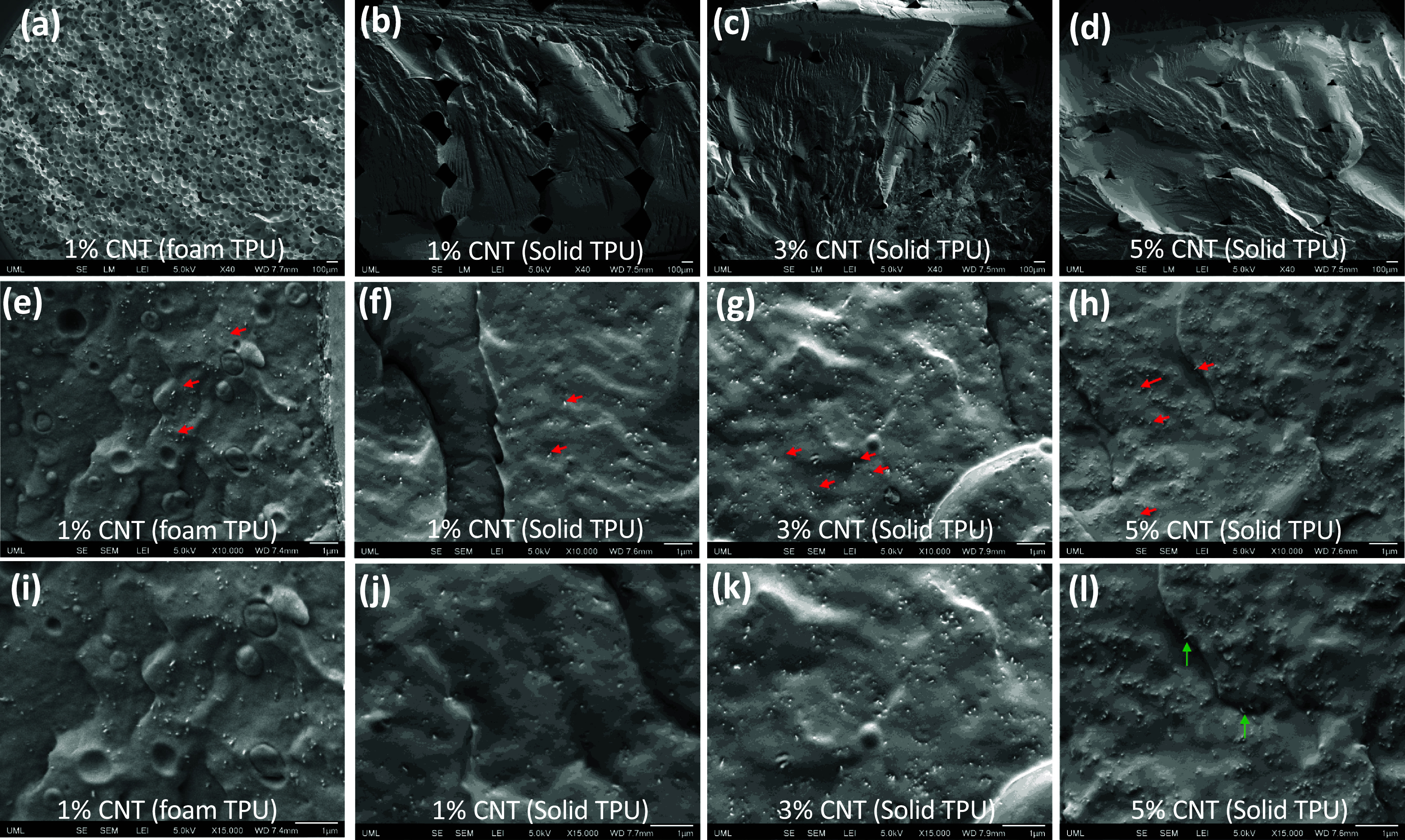
SEM pictures captured for foam TPU with 1% CNT and solid TPU with different percentages (1%, 3%, and 5%) of CNT: (a)–(d) captured at low magnification; (e)–(h) captured at medium magnification; (i)–(l) captured at high magnification.

Scanning electron microscopy (SEM) images were captured to explain the reason behind the optimum CNT content leading to the maximum output (figure 8). The figure shows the SEM micrographs of CNT/TPU solid samples. The micrographs were taken on the cryo-fractured surfaces, revealing the cross-section of each raster. A thin gold layer was sputter-coated onto the fracture surfaces for 180 s using a Leica SCD500 sputter coater. Field emission SEM (JEOL JSM 7410 F) was used to capture the images.

To examine the effect of CNT on micro-voids and the capacitance, we present the SEM images of foam and solid CNT/TPU composites with different CNT content, taken under the low magnification mode in figures 8(a)–(d). The interlayer voids are observed across all the solid samples. The solid CNT/TPU with 1% content of CNT showed the largest interlayer gaps, while the 3% and 5% CNT samples showed significantly smaller interlayer gaps inside the structure, and this can be because of the CNT effect on the rheological and heat transfer behavior of the TPU matrix. Foam CNT/TPU with 1% content of CNT exhibits a highly porous structure. Foaming transforms the large interlayer voids between printed rasters into smaller, more controlled voids throughout the structure [49]. The presence of these voids increases the overall capacitance and effective relative permittivity for the same thickness as the solid prototype. The larger voids may also create more internal friction during contact and separation, and the charge density increases. Due to these increases, the instantaneous current and power will increase.

SEM images with higher magnifications were used to study agglomeration. Medium-magnification SEM images of CNT/TPU composites containing foam 1% CNT and solid 1%–5% CNT are depicted in figures 8(e)–(h), respectively. The red arrows highlight the dispersion of the CNTs inside the TPU matrix. No significant CNT agglomeration is observed in the images. CNT orientation appears in some areas to align with the raster orientation, and they are locally concentrated around the cell walls in the microcellular structure. Figures 8(i)–(l) present high-magnification SEM images of the CNT/TPU composites. The green arrows indicate that a single CNT pulls out, confirming that the dispersion is relatively good. The visibility of the distinct nanotubes suggests that single CNTs are well dispersed and distinguishable.

**Figure 9.**
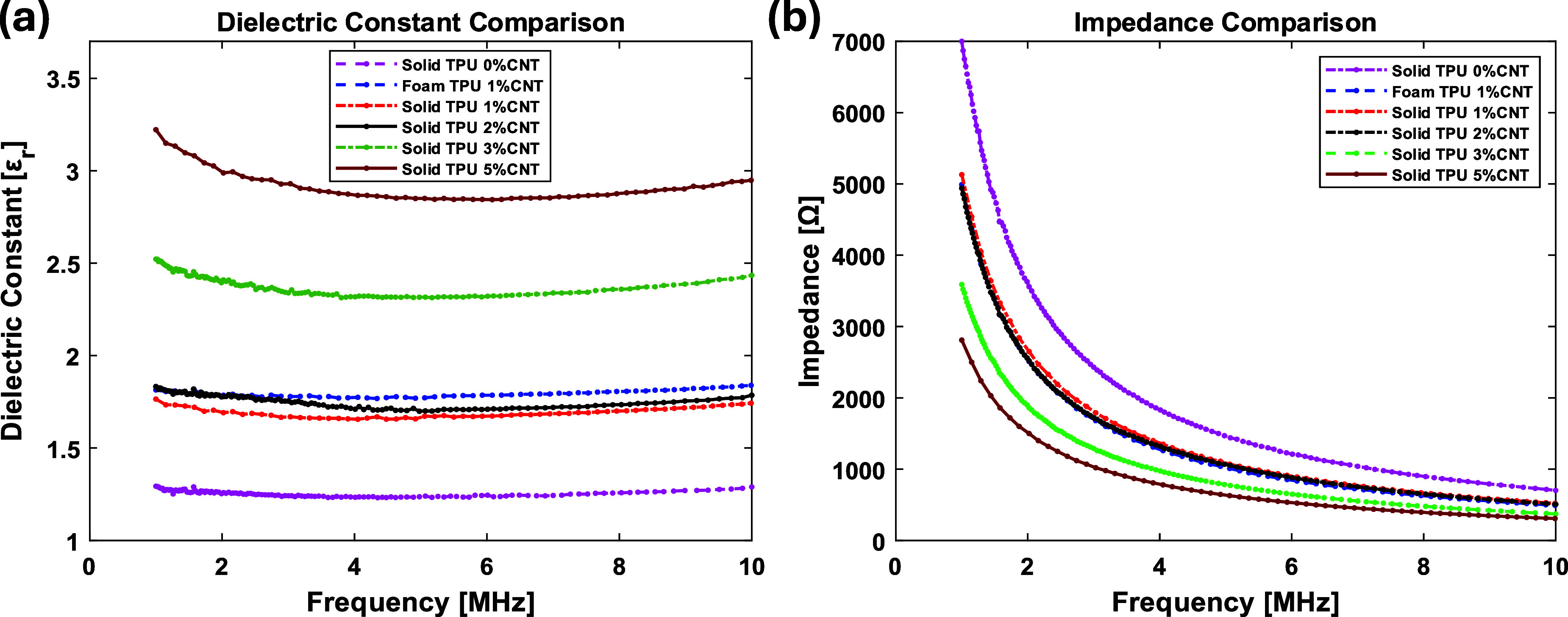
(a) Shows the calculated values of the dielectric constant for the same samples; (b) shows the measured impedance of some CNT/TPU-based TENG samples with different CNT contents.

To further understand the enhancement of the foam CNT/TPU-based TENG’s power output, we investigated each sample’s dielectric behavior. The foam CNT/TPU-based TENG has a highly porous structure, which increases the dielectric constant, and that would increase the power output [59]. We conducted impedance measurements using a Keysight ENA network analyzer for foam and solid samples across different CNT contents, as shown in figure 9(b). From these impedance values, we calculated the dielectric constant as shown in figure 9(a). Our measurements confirm that the foam TPU with 1% CNT exhibits a higher dielectric constant compared to its solid counterpart, aligning with the observed increase in output. The data show that impedance decreases with increasing CNT content, from which we derived the dielectric constants. It is deduced that increasing CNT concentration raises the dielectric constant. However, beyond 1% CNT, the formation of voids is suppressed, as verified by the SEM images discussed earlier. This reduction in porosity leads to a decrease in output despite the higher CNT content. It is because microvoids increase the charge density during compression and release, leading to higher current and power.

**Figure 10.**
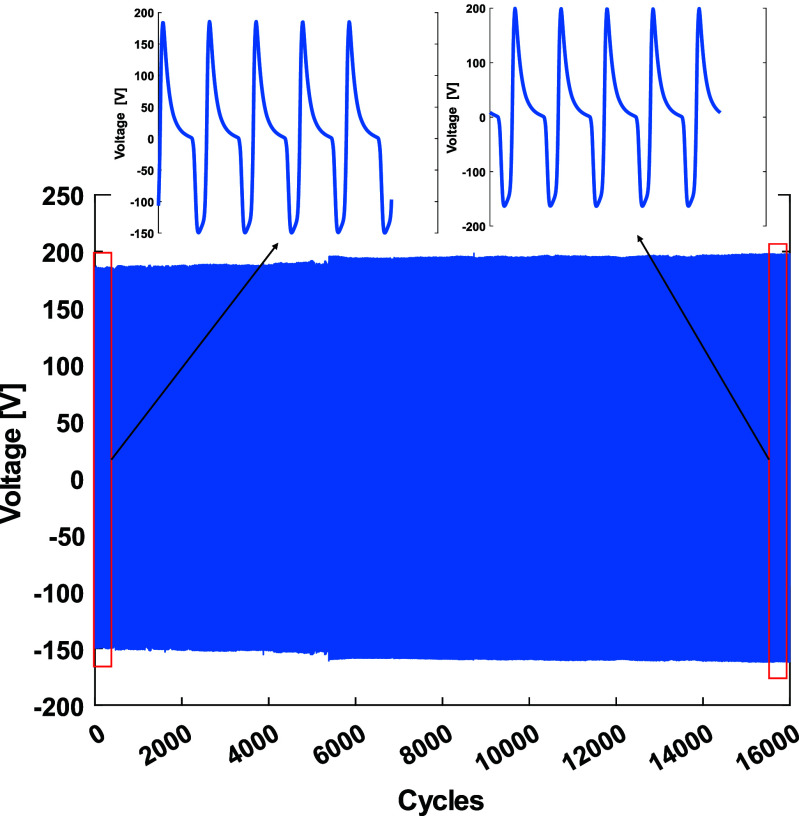
Durability test of foam CNT/TPU for 16 000 cycles.

To evaluate the mechanical durability of the foam CNT/TPU-based TENG with 1% CNT concentration, a cyclic load test was conducted over 16 000 cycles under a repeated compressive load of 2100 N at a frequency of 1 Hz (figure 10). As shown in the figure, the output voltage remained stable initially, then increased by approximately 30 peak-to-peak volts after around 5000 cycles, and subsequently stabilized for the remainder of the test. This voltage increase is attributed to a slight reduction in foam thickness due to plastic deformation of the TPU around the 5000-cycle mark, which increases the gap, reduces capacitance, and thus enhances voltage output. These results demonstrate the electrical stability of the foam CNT/TPU-based TENG over extended use, supporting its potential for long-term energy harvesting applications. However, it is important to note that some samples experienced tearing during testing, indicating that the mechanical durability of 3D-printed TPU requires further investigation.

**Figure 11.**
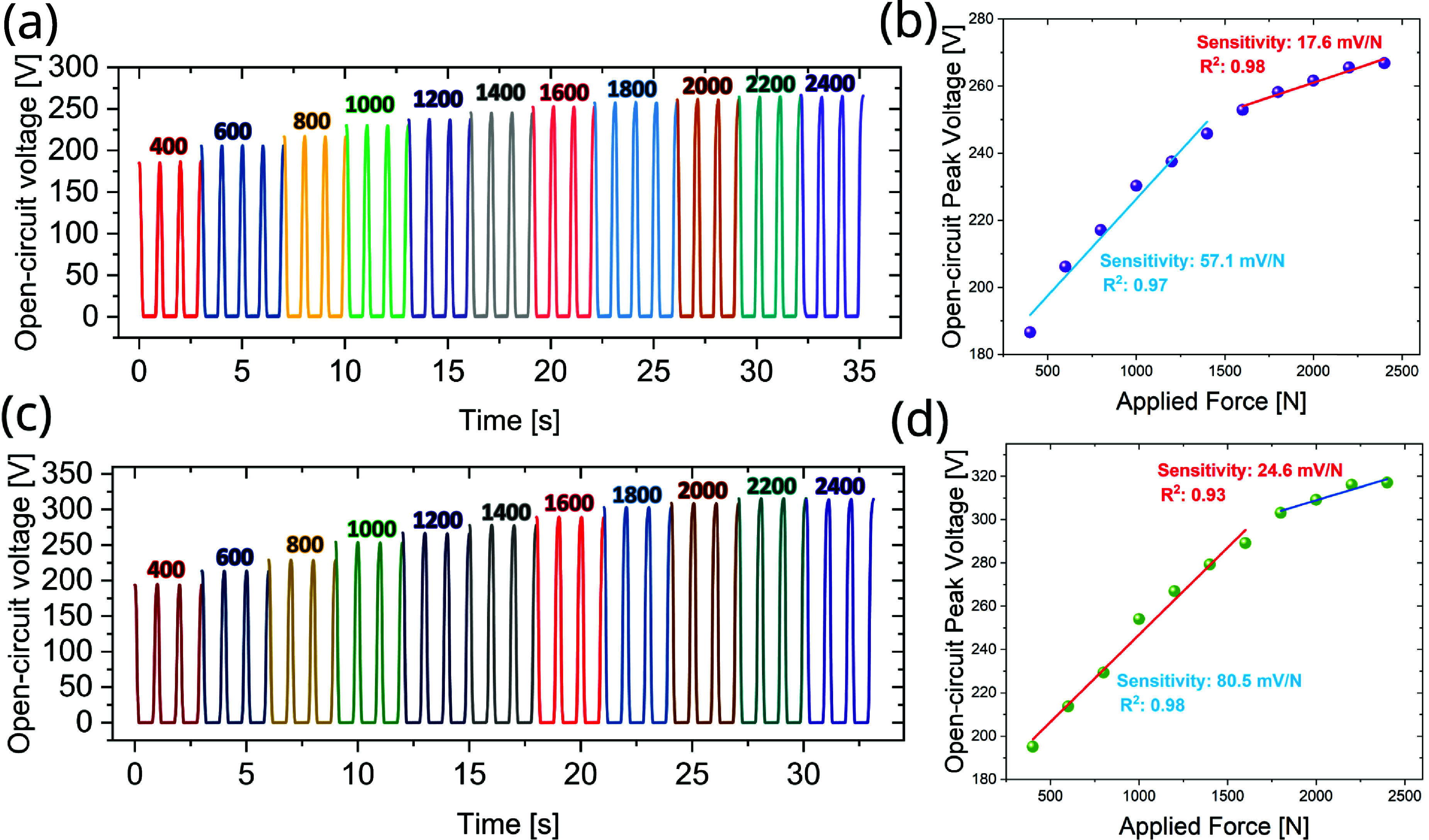
(a) and (c) Open circuit peak-to-peak voltages with different applied forces for solid and foam CNT/TPU-based TENG, respectively, at 1% CNT content.(b) and (d) The sensitivity to the applied force for the solid and foam CNT/TPU, respectively, at 1% CNT content.

Furthermore, we examined the RMS voltage response to various ranges of axial compression forces (ranging from 400 N to 2400 N) to evaluate the sensitivity of the TENG as a force sensor for both solid 1% CNT/TPU (figures 11(a) and (b)) and foam 1% CNT/TPU (figures 11(c) and (d)). The linear relationship between the peak-to-peak open circuit voltages and different applied forces is shown in parts (b) and (d) in two distinct regions. The two sensors exhibit high sensitivity for forces below 1600 N of 57.1 mV N^−1^ ($R^2 = 0.97$) and 80.5 mV N^−1^ ($R^2 = 0.98$) for the solid and foam prototypes, respectively. The higher sensitivity in this region is due to the large deformation of the TKR package and embedded TENG sensors. The sensitivity drops to 17.6 mV N^−1^ ($R^2 = 0.98$) and 24.6 mV N^−1^ ($R^2 = 0.93$) for the solid and foam sensors beyond 1600 N. The drop is likely due to nonlinear behavior in the spring, which causes a reduction in the voltage increase per unit force. These results show the proposed CNT/TPU-based TENG’s capability for precise force sensing, with superior sensitivity at lower forces and stable performance across a wide force range.

**Figure 12.**
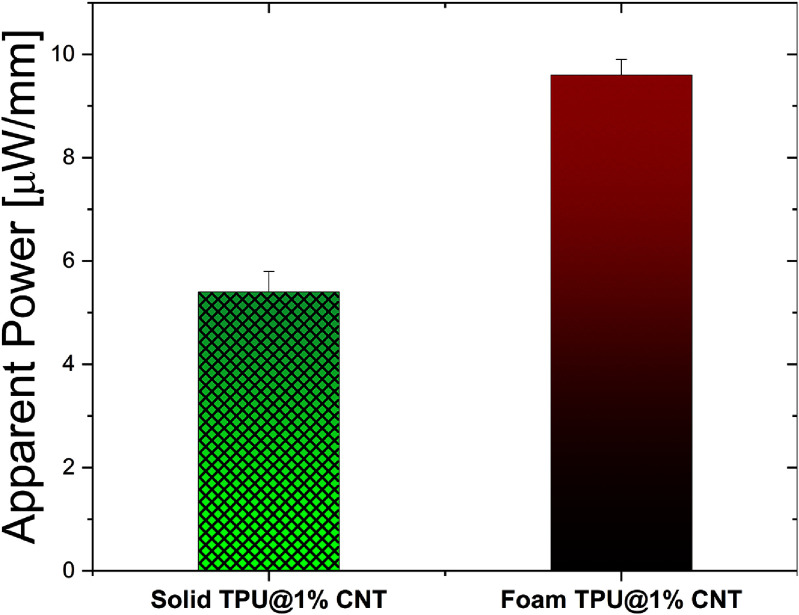
Normalized apparent power by thickness output comparison between solid and foam CNT/TPU at 1% CNT.

In summary, the foam CNT/TPU-based TENG exhibits higher power output than solid TPU, with thinner foamed TPU layers enhancing charge storage capacity and boosting power generation. A CNT concentration of 1% remains optimal for triboelectric energy harvesting, as higher concentrations do not improve performance. The highest apparent output powers for solid and foam CNT/TPU-based TENG at 1% concentration are presented in figure 12, with both samples tested under identical conditions and nominal thicknesses. The measured samples’ thicknesses (using a digital caliper) for the solid and foam ones were 0.76 mm and 0.72 mm, respectively. The samples’ output powers were normalized to the thickness, where the solid sample normalized power is 5.4 $\mu\mathrm{W\,mm}^{-1}$, and for the foam sample. The superior charge density of foam TPU is attributed to internal pore surfaces generating charges upon compression, leading to greater charge production than solid TPU.

Table 2 compares the output of the 3D-printed CNT/TPU prototype with that of other transducers reported in the literature. The output of the presented prototype is relatively lower compared to some piezoelectric-based harvesters. However, the CNT/TPU may not suffer from toxicity introduced by the lead material inside PZTs. It is also important to note that the output values are directly influenced by the applied load magnitude. Among the references reviewed, only [46] reports the output within the physiological load range relevant to the knee joint, the primary focus of this study. Some references, such as [62] report a high output of 30 *µ*W cm^−2^, but for tire pressure application, where the loads are much larger.

**Table 2.** Summary of different transducer outputs.

S. no.	Materials	Output performance	Application	reference
1	8-layer stacked PVDF films	52 $\mu\mathrm{W\,cm}^{-2}$	Shoe sole	[60]
2	PZT inside Polyamide	1.2 $\mu\mathrm{W\,cm}^{-2}$	Pacemakers	[61]
3	Urethane/PZT-5A	30 $\mu\mathrm{W\,cm}^{-2}$	Tire pressure	[62]
4	BaTiO_3_/dopamine/silicon rubber	1.271 $\mu\mathrm{W\,cm}^{-2}$	Knee implant	[46]
5	Ag NW/CB/TPU	0.43 $\mu\mathrm{W\,cm}^{-2}$	Human–machine interfaces	[39]
6	TPU/mica nanofiber	0.146 mW cm^−2^	Wearable motion sensing	[63]
7	TPU/PLA/Carbon	5.838 $\mu\mathrm{W\,cm}^{-2}$	Wearable sensors for athlete	[64]
8	CNT/TPU	0.43 $\mu\mathrm{W\,cm}^{-2}$	Knee implant	This work

The findings presented here contribute valuable insights toward the advancement of self-powered sensor technologies for TKR implants. While the complete housing package has a total thickness of 19 mm, the energy harvester layers themselves are only 3–5 mm thick. This increased package thickness was intentionally selected to facilitate experimental testing. Future iterations will leverage advanced fabrication techniques and optimized packaging to significantly reduce thickness and better align with clinical requirements.

Although the foam CNT/TPU harvester generates a relatively modest maximum power output of approximately 6.9 *µ*W, this is sufficient to operate low-power components such as the ADC circuit, which our group has demonstrated consumes about 5.35 *µ*W [58]. For wireless transmission of load-sensing data, we plan to integrate an inductive link in future designs. In this system, an external primary coil transmits electromagnetic energy to a secondary coil embedded in the implant. Importantly, our design does not rely on continuous inductive powering, which can be a major limitation. Instead, the sensor operates continuously on harvested power, with the inductive link used briefly—less than a minute per day—solely for data transmission.

## 6. Conclusion

A 3D-printed CNT/TPU-based TENG for self-powered load sensing in knee implants is presented. The effect of CNT percentage on the triboelectric performance of TPU for TKR application is evaluated. Since the knee is among the body joints that receive the most amount of load, transducers such as TENGs have a great potential for converting biomechanical energy to electricity for self-powered joint load monitoring. The optimal percentage for CNT to achieve the highest power output is reported. The 1% CNT/TPU demonstrated the highest power output, with solid TPU generating 5.4 $\mu\mathrm{W\,mm}^{-1}$ at 2100 N and foam TPU reaching 9.6 $\mu\mathrm{W\,mm}^{-1}$ across an area of 15.9 $\mathrm{cm}^{2}$. The optimal resistances for the prototypes were 1.6 GΩ and 0.9 GΩ, respectively. This power output is sufficient to operate power management and ADC circuits, demonstrating the feasibility of using the proposed TENG for self-powered sensing and energy harvesting in TKR implants. The TENG exhibited a linear voltage-force relationship, enabling its force-sensing capability. Additionally, the dielectric constant of the CNT/TPU composite increased with CNT concentration. Durability testing of the foam CNT/TPU TENG under 16 000 loading cycles showed stable voltage output, confirming its potential for long-term use. These results show that the proposed 3D printed CNT/TPU-based TENG could offer a feasible solution for real-time monitoring in TKR implants.

## Data Availability

All data that support the findings of this study are included within the article (and any supplementary files).
